# Effect of Perceived Negative Workplace Gossip on Employees’ Behaviors

**DOI:** 10.3389/fpsyg.2018.01112

**Published:** 2018-07-12

**Authors:** Ming Kong

**Affiliations:** School of Management, Shandong University, Jinan, China

**Keywords:** perceived negative workplace gossip, in-role behavior, organizational citizenship behavior, organization-based self-esteem, perceived insider status, hostile attribution bias

## Abstract

Negative workplace gossip generates social undermining and great side effects to employees. But, the damage of negative gossip is mainly aimed at the employee who perceived being targeted. The purpose of this study is to develop a conceptual model in which perceived negative workplace gossip influences employees in-role behavior and organizational citizenship behavior differentially by changing employees’ self-concept (organizational-based self-esteem and perceived insider status). 336 employees from seven Chinese companies were investigated for empirical analysis on proposed hypotheses, and results show that: (1) Perceived negative workplace gossip adversely influences employees’ IRB and OCB. (2) Self-concept (OBSE and PIS) plays a mediating role in the relationship between perceived negative workplace gossip and employees’ behaviors (IRB and OCB). (3) Employees’ hostile attribution bias moderates the relationship between perceived negative workplace gossip and self-concept (OBSE and PIS); and also moderates the mediating effect of self-concept (OBSE and PIS) on the relationship between perceived negative workplace gossip and employees’ behaviors (IRB and OCB). Thus, our findings provide deeper insights into the potential harmful effects of gossip. In addition, we help to explain the underlying mechanism and boundary condition of these effects.

## Introduction

What new “gossip” have you heard in tea room? Does your colleague start with the sentence: “Have you heard that?”? – “Gossip” exists anywhere people live, and definitely occurs in offices hotly contested by modern people to work in. Scholars indicate that 14% workplace coffee-break chat is actually gossip and about 66% of general conversion between employees is related to social topics concerning talk about other colleagues (Cole and Dalton, 2009). In organizations, gossips serve as a major tool to strengthen informal employee relationship (Noon and Delbridge, 1993; Dunbar, 2004; Kniffin and Wilson, 2005). Such *“negative, informal and evaluative talk in an organization about another member of that organization who is not present” is called negative workplace gossip* (de Gouveia et al., 2005; Chandra and Robinson, 2010; Wu et al., 2016).

Most prior studies focused on negative workplace gossip have made the topic gradually become a hotspot in organizational behavior field overseas (Foster, 2004; Waddington and Michelson, 2007; Feinberg et al., 2014; Wu et al., 2016; Brady et al., 2017). Existing studies have probed into such antecedent variables of negative workplace gossip as individual factors (value, organization’s hierarchy, etc.) (Gluckman, 1968; Ellwardt et al., 2012) and organizational factors (organization’s integrity, power structure, etc.) (Noon and Delbridge, 1993; Baumeister et al., 2004). Some scholars believe that negative workplace gossip is a type of social undermining for employees (Duffy et al., 2002), and employees surrounded by such negative gossips will find it hard to trust others or establish good cooperative relationship (Aquino and Thau, 2009); meanwhile, negative workplace gossip can result in great side effects to employees (Baumeister and Leary, 1995; Ellwardt et al., 2012), such as lowering employees’ work efficiency and job satisfaction, etc. (Michelson and Mouly, 2000; Greengard, 2001) and bring about more harm than good to their team (Elias and Scotson, 1994). In short, these research achievements are just pioneering, and more future efforts are necessary. Since negative workplace gossip exists everywhere, cannot be eliminated and exerts wide influence on organizations, will it impact employees’ behaviors?

Scholars have urged greater attention to the psychological and attitudinal outcomes of workplace gossip on the gossiper (e.g., Waddington and Fletcher, 2005; Farley et al., 2010), such as gaining personal power and reputation (McAndrew et al., 2007), but to our knowledge, few studies have examined negative workplace gossip from the target’s perspective (see Ellwardt et al., 2012). Indeed, we know little about how the perception of being targeted by negative workplace gossip will influence one’s work-related behaviors (Wu et al., 2016) and, specifically, the process through which perceived negative workplace gossip might influence in-role behavior (IRB) and organizational citizenship behavior (OCB).

The self-evaluation perspective (Gecas, 1982; Jussim et al., 1992; Shrauger and Schoeneman, 1999) may be useful for understanding the effects of perceived negative workplace gossip. From this perspective, it is believed that individuals take their own value and role in organizations as one part of self-concept (Gecas, 1982). Negative workplace gossip refers to negative comments about one employee made by other colleagues. It is one of external information sources of self-evaluation. We believe that, employees who perceived negative workplace gossip are likely to combine outside negative evaluation into their own self-evaluation, which negatively impacts their behaviors (Fleith et al., 2002). Secondly, employees tend to integrate outside information into self-evaluating (Swann et al., 1994). Organizational-based self-esteem (OBSE) and perceived insider status (PIS) are related to external evaluation, and negative workplace gossip just strengthens external negative evaluation on employees and lowers employees’ OBSE and PIS. According to self-verification theory, employees’ negative self-evaluation can exert negative influence on their workplace behaviors (Fleith et al., 2002). Hence, withholding OBSE and PIS will weaken employees’ IRB and OCB. Nevertheless, the influence of individual factors also affects the function of perceived negative workplace gossip. For example, individual traits may affect function direction. Hostile attribution bias is a type of external attribution inclinations, which is an individual trait. Therefore, this paper intends to explore the moderating effect of hostile attribution bias (Adams and John, 1997) between perceived negative workplace gossip and OBSE, and aims to comprehensively analyze the function boundary of perceived negative workplace gossip.

In conclusion, by examining the moderated mediation model, we make three contributions to the negative gossip and positive behavior literature. First, whereas prior research has primary attention to the psychological and attitudinal outcomes of workplace gossip on the gossiper (e.g., Waddington and Fletcher, 2005; Farley et al., 2010), we examined negative workplace gossip from the target’s perspective and enriches researches on negative workplace gossip, especially in Chinese cultural background. Second, we identify OBSE and PIS as the mediators and hostile attribution bias as the moderator explaining why and how negative workplace gossip affects IRB and OCB. We not only test the direct influence of negative workplace gossip on IRB and OCB, but also examine the function mediating mechanism and boundary condition of negative workplace gossip. Third, our study has important implications for organizations. Negative workplace gossip can significantly withhold OBSE and PIS, and then reduce employees’ positive workplace behaviors (IRB and OCB). Therefore, organizations should take measures to handle negative workplace gossip.

## Theory and Hypotheses

### Perceived Negative Workplace Gossip

*Annals of the Kingdoms in the East Zhou Dynasty* summarizes gossip’s harm as, “little gossip’s harm is about good/ill luck of one person, and great harm is about rise/decline of one country.” Negative workplace gossip is characterized by universality, perniciousness, and richness. (1) Universality refers to the fact that workplace negative gossip widely exists in organizations, cannot be completely eliminated (Noon and Delbridge, 1993) and has a very high frequency of occurrence (Dunbar et al., 1997). Near 65% speeches are gossips (Dunbar, 2004), which is also true for organizations (Foster, 2004). Previous research proved that people attach more attention to negative information than positive and neutral information (Barkow, 1992; Davis and McLeod, 2003; Baumeister et al., 2004). (2) Perniciousness is mainly reflected by the impact on gossip target and organizational atmosphere. Negative workplace gossip generates social undermining (Duffy et al., 2002) and great side effects to employees (Baumeister and Leary, 1995; Ellwardt et al., 2012). Under many circumstances, negative workplace gossip is always used by many dishonest people as a tool in the organization’s political struggle (McAndrew et al., 2007), thus giving the previously stable interpersonal relationship the color of suspicion, in-fighting and tension, bringing the order in chaos, and making employees in such an organization captives of gossip. It destroys unity, makes everyone jittery, and weakens mutual trust, exerting negative influence on the employees’ working attitude and behavior (Aquino and Thau, 2009; Chandra and Robinson, 2010). (3) Richness means that workplace negative gossip contains rich information sources, and can reveal many problems in enterprise management (Waddington and Michelson, 2007); however, after being transmitted, the contents and property of negative gossip may suffer great changes. As the gossip spreads farther, it will become more twisted and even more malicious (Bok, 1989). To the end, targeted research and systematic countermeasures analysis are needed.

Negative gossip refers to negative information about an employee that others talk about at his/her back or disseminate maliciously and the employee can experience in the workplace. It is a type of common social behavior (Ellwardt et al., 2012; Grosser et al., 2012) and constitutes an important part of organizational life (Kniffin and Wilson, 2010). But, the damage of negative gossip is mainly aimed at the employee who perceived being targeted (Ellwardt et al., 2012). Therefore, it is meaningful to explore the mechanism of negative workplace gossip from the “perceived” perception.

Investigation on it can be traced back to symbolic interactionist views (Mead, 1934) and enhanced by self-verification and self-evaluation view (Swann et al., 1992). Formation of individual’s self-concept stems from interpersonal interaction. That’s to say, an individual’s self-evaluation is mainly derived from others’ evaluation (e.g., the Looking-Glass Self; Cooley, 1902). As previously mentioned, negative workplace gossip means undermining to the employee who perceived being targeted (Duffy et al., 2002), and privacy disclosure will make employees perplexed by negative gossip embarrassed, thus adding psychological and physical pressure; and under such circumstances, these employees will be forced to spend a large amount of time and efforts on clarification of negative gossips (Chandra and Robinson, 2010). Existing studies have proven that negative workplace gossip can lower employees’ job satisfaction and production efficiency, thus exhausting emotions and increasing such phenomena as dimission, asking for leave, etc. (Michelson and Mouly, 2000; Greengard, 2001). Thus, it can be seen that perceived negative workplace gossip indeed exerts strong negative influence on individuals (Baumeister and Leary, 1995; Ellwardt et al., 2012).

### Perceived Negative Workplace Gossip and Employees’ Behaviors

Self-evaluation (Swann et al., 1992) refers to assessment made by individuals on their own thinking, abilities, levels, etc., and is a key component of self-adjustment mechanism. Moreover, self-evaluation is based on a certain amount of information, collected via many channels, including self-evaluation, other-evaluation, comparison, etc. As a kind of unhealthy interpersonal interaction experience, negative workplace gossip can exert significant influence on employees’ behaviors (Wu et al., 2016). As such, this study explores employees’ behaviors from two dimensions: IRB and OCB. IRB is one part of employees’ work, and clearly expected, evaluated, and awarded by organizations (Bargh et al., 1996). OCB is the employees’ conscientious behavior not clarified or directly stipulated by formal reward system, beneficial to improving the effectiveness of organizational function (Bateman and Organ, 1983; Williams and Anderson, 1991).

According to self-verification theory, perceived negative workplace gossip exerts negative influence on employees’ behaviors: (1) Negative workplace gossip plays an important role in forming and strengthening employees’ self-evaluation. To be specific, employees who perceived negative workplace gossip tend to integrate negative external evaluation and thus negatively evaluate self. Studies have proven that negative self-evaluation can exert negative influence on employees’ behaviors (Fleith et al., 2002). (2) The victims regard negative workplace gossip as experiences of undesirable interpersonal interaction, relating to negative evaluation and always leading to enormous psychological burden and psychological insecurity. Studies have shown that employees with psychological burden and psychological insecurity also bear negative impact on their workplace behaviors (Probst et al., 2007). (3) In an organization inundated with negative gossips, people will lose trust on others, thus leading to mutual suspicion, hostility, and noncooperation. Under the circumstances, employees will be trapped in such negative induced emotions as anxiety, disappointment, anger, depression, etc. (Hobfoll, 1989; Agnew, 1992), and always suffer from emotion exhaust (Michelson and Mouly, 2000; Greengard, 2001), all the negative reaction from which can negatively impact employees’ behaviors (Fredrickson, 1998).

Some scholars think that gossip shall be re-conceptualized into job-related gossip (JRG) and non-job-related gossip (NJG), and further found that compared to NJG, JRG is more likely to predict employees’ behaviors (Kuo et al., 2015). Specially, in our study, IRB and OCB are all related to employees’ job, and more likely to be influenced by negative workplace gossip. Based on this, this study proposes the following hypotheses:

H1a: Perceived negative workplace gossip negatively influences employees’ in-role behavior.
H1b: Perceived negative workplace gossip negatively influences employees’ organizational citizenship behavior.

### The Mediating Effect of Self-Concept

Self-concept is a construct in social psychology and with some multidisciplinary developments (Gecas, 1982). Epstein (1973) defines self-concept as a theory person holds about himself experiencing and functioning interact with the outside world, from a perspective of attribution, emphasizing knowledge and beliefs an individual owns. Self-concept is divided as self-conception and self-evaluation, from structural content aspect and evaluative and affective aspect, respectively (Gecas, 1982). Individual’s self-concept appears as different status under different social contexts. Some researches investigate the reconceptualization of self-concept within group situations (Alexander and Knight, 1971; Webster and Sobieszek, 1974), which can be regarded as self-concept based on organization. Thus an employees’ self-concept in an organization can be divided as organization-based self-esteem from evaluative aspect and PIS from structural aspect (Chen and Aryee, 2007).

To be specific, OBSE reflects the value of employees sensed in their organization, and refers to the satisfying degree of internal demand experienced by employees, and also self-concept and self-evaluation developed by employees (Pierce et al., 1989; Pierce and Gardner, 2004). PIS is the extent in which an employee’s perception of membership in a certain organization, i.e., being inside or in the periphery of the organization (Stamper and Masterson, 2002). According to self-verification theory, employees with high OBSE and PIS can perceive their important, significant, and valuable role in an organization, and make more efforts in improving IRB and OCB (Pierce and Gardner, 2004).

First, perceived negative workplace gossip can affect employees’ self-concept (OBSE and PIS). Based on self-evaluation perspective, individuals evaluate the value of themselves in their organizations based on external information. OBSE refers to the satisfying degree of internal demand experienced by employees by playing a role in their organizations, and also self-evaluation developed by employees in specific organizations (Pierce et al., 1989; Pierce and Gardner, 2004). And PIS is the extent in which an employee’s perception of membership in a certain organization, i.e., being inside or in the periphery of the organization (Stamper and Masterson, 2002). Meanwhile, employees’ OBSE and PIS is related to external evaluation, and negative workplace gossip just strengthens external negative evaluation on employees and lowers employees’ OBSE and PIS. Since employees often tend to integrate social evaluation by others (such as leaders, colleagues, or subordinates) into their own self-evaluation, interpersonal interaction signal in an organization is one important factor affecting OBSE and PIS (Korman, 1970). Negative social evaluation by others (for example, poor morality, weak ability, low contribution, etc.) will reduce employees’ OBSE and PIS (Korman, 1970, 1976).

Second, self-concept can affect employees’ behaviors (Van Dyne and Pierce, 2004). Self-consistency considers that employees endeavor to maintain consistency of self-cognition with attitudes and behaviors (Korman, 1970). To be specific, employees with high OBSE and PIS believe in their value in organizations, and always show positive work attitudes and behaviors in order to sustain positive self-cognition; on the contrary, employees with low OBSE and PIS cannot obtain value identification in organizations (with negative self-cognition), and always demonstrate negative work attitudes and behaviors (Pierce and Gardner, 2004).

Thus, the following hypotheses are made:

H2a: Self-concept (OBSE and PIS) mediates the relationship between perceived negative workplace gossip and employees’ in-role behavior.
H2b: Self-concept (OBSE and PIS) mediates the relationship between perceived negative workplace gossip and employees’ organizational citizenship behavior.

### The Moderating Effect of Hostile Attribution Bias

Hostile attribution bias means that individuals are inclined to give hostile explanations to an equivocal context (Adams and John, 1997), which is a type of external attribution inclinations. Individuals with high hostile attribution bias are very sensitive to others’ attitudes and behaviors, tend to give hostile explanations to others’ attitudes and behaviors if they are unable to pinpoint others’ motive behind, and will also be apt to treat it as a hostile signal even when others’ words or behaviors is not “hostile” (Tedeschi and Felson, 1994). They attribute failure to such external factors as environment, others, etc., and exaggerate their contribution (Campbell et al., 2000). Specially, hostile attribution bias plays a greater role in negative contexts (Thomas and Pondy, 1977), and individuals with high hostile attribution bias bear stronger negative psychological feelings in such negative contexts. Therefore, in front of strong perceived negative workplace gossip, employees with high hostile attribution bias will consolidate its negative influence on their psychology, which grows stronger and stronger, obviously withholding self-concept (OBSE and PIS).

Accordingly, this study proposes the following hypothesis:

H3: Hostile attribution bias moderates the relationship between perceived negative workplace gossip and employees’ self-concept (OBSE and PIS), namely, the higher the hostile attribution bias, the stronger the relationship between perceived negative workplace gossip and self-concept (OBSE and PIS).

All above, Hypothesis 2 implies that the mechanism of perceived negative workplace gossip on employees’ behaviors is “self-concept,” namely, perceived negative workplace gossip can lower the level of OBSE and PIS, thus decreasing IRB and OCB. Hypothesis 3 indicates that hostile attribution bias moderates the relationship between perceived negative workplace gossip and self-concept (OBSE and PIS), specifically, strengthens the negative relationship between perceived negative workplace gossip and self-concept (OBSE and PIS). Logically, employees perceived by negative workplace gossip with high hostile attribution bias will strengthen the negative role on OBSE and PIS, resulting in less IRB and OCB.

In summary, in combination with Hypotheses 2 and 3, the study further proposes two moderated mediation models. We further propose that employees’ hostile attribution bias moderates the mediating effect of OBSE and PIS in the relationship between perceived negative workplace gossip and employees’ IRB/OCB. To be specific, employees with high hostile attribution bias will consolidate the impact of negative information on themselves while attacked by negative workplace gossip, and their OBSE and PIS will be reduced as well, further lowering their IRB and OCB. That is to say, the indirect negative influence of negative workplace gossip on IRB and OCB is strengthened through the mediating effect of OBSE and PIS. On the contrary, employees with low hostile attribution bias will weaken and even “shield” the impact of negative information on themselves in the face of negative gossip due to the weak impact of negative information or context, and the impact of negative workplace gossip on their OBSE and PIS will be reduced, further affecting their workplace behavior.

H4a: Hostile attribution bias moderates the mediating effect of self-concept (OBSE and PIS) between negative workplace gossip and employees’ in-role behavior: the higher the hostile attribution bias, the greater the mediating effect of OBSE and PIS between negative workplace gossip and employees’ in-role behavior.
H4b: Hostile attribution bias moderates the mediating effect of self-concept (OBSE and PIS) between negative workplace gossip and employees’ organizational citizenship behavior: the higher the hostile attribution bias, the greater the mediating effect of OBSE and PIS between negative workplace gossip and employees’ organizational citizenship behavior.

**FIGURE 1:**
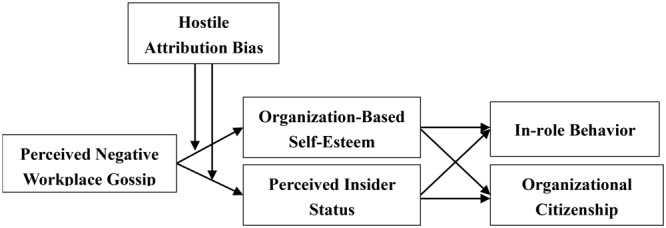
Summary model of hypothesized relationships.

A graphical representation of the study goals is illustrated in **Figure 1**.

## Materials and Methods

### Procedure

The study was approved by School of Management of Shandong University in China. All participants provided written informed consent. We selected full-time employees from traditional work teams of seven Chinese companies in diverse industries and with various job types to increase external validity of proposed relationships. The process was completed with support from human resources departments of participating enterprises. In order to minimize common method variance (Podsakoff et al., 2003), two questionnaire surveys were conducted successively with 2 weeks between: objects in the first survey (T1) were employees, and research content included employees’ personal background information, perceived negative workplace gossip, OBSE, and PIS; objects in the second survey (T2) were employees’ leaders, and research content included employees’ IRB and OCB; objects in the third survey (T3) were employees, and research content included employees’ hostile attribution bias.

With regard to ethical standards for research, the study adhered to the latest version of the Declaration of Helsinki revised in Fortaleza (World Medical Association [WMA], 2013).

### Participants

In T1, 450 questionnaires were distributed to employees. Among them, 383 (85.1%) valid questionnaires were collected. Two weeks later, T2 was conducted with the above 383 employees’ leaders. Among 383 dyads, 341 (89.0%) valid questionnaires were collected. Two weeks later, T3 conducted with the above 341 employees. Finally we had valid questionnaires of 336 dyads: 43.2% employees were male and the average age of all samples was 28.94 years old (*SD* = 2.94). Employees had, on average, 6.08 years of work experience (*SD* = 2.75) and 2.23 years of working with their direct leaders (*SD* = 1.69).

### Measures

In this study, measuring scales all came from leading international journals and boast good psychometric property. We followed the commonly used back-translation procedure proposed by Brislin (1986) to translate them into Chinese. Unless otherwise indicated, all following measures were rated by employees and their direct leaders on a 5-point Likert type scale ranging from strongly disagree (1) to strongly agree (5).

Perceived negative workplace gossip: A three-item scale developed by Chandra and Robinson (2010) was used to measure perceived negative workplace gossip. It was ranked by employees and a sample item included “In the past 6 months, others (e.g., coworkers and/or supervisors) communicated damaging information about me in the workplace.” (α = 0.80).

Organizational-based self-esteem was ranked by employees with 10 items on a five-point scale developed by Pierce et al. (1989). A sample item included “I am taken seriously around here” (α = 0.92).

Perceived insider status was measured with six items scale developed by Stamper and Masterson (2002). Items samples are “I feel very much a part of my work organization” and “I feel I am an insider in my work organization.” The scale score was calculated by summing the individual item scores (α = 0.94).

Hostile attribution bias was measured with six-item scale developed by Adams and John (1997). It was ranked by employees and a sample item included “A person is better off if he/she doesn’t trust anyone” (α = 0.79).

In-role behavior and OCB were measured with 15 items on a five-point scale developed by Williams and Anderson (1991). It was ranked by employees’ leader and a sample item included “This employee performs tasks that are expected of me” and “This employee conserves and protects organizational property,” respectively (α = 0.88, 0.88).

Besides above key variables, this study took employees’ age, gender, education, seniority and experience, and years of working with their leaders as control variables.

## Analysis and Results

### Confirmatory Factor Analysis

We first sought to examine the discriminant validity of the measure and conducted a confirmatory factor analysis (CFA) with LISREL 8.8 to examine the distinctiveness of the multi-item variables in the study. As shown in **Table 1**, the hypothesized five-factor model [χ^2^(194) = 538.07, CFI = 0.95, TLI = 0.92, IFI = 0.95, SRMR = 0.05, RMSEA = 0.07] fits the data better than other models, supporting the distinctiveness of variables in this study.

**Table 1:** Results of confirmatory factor analyses.

Model	χ^2^	*df*	CFI	TLI	IFI	SRMR	RMSEA
Six-factor model	**538.07**	**194**	**0.95**	**0.92**	**0.95**	**0.05**	**0.07**
Five-factor model^a^	1255.47	220	0.86	0.84	0.86	0.09	0.12
Four-factor model^b^	1850.74	224	0.78	0.76	0.78	0.11	0.15
Three-factor model^c^	2157.12	227	0.74	0.71	0.74	0.13	0.16
Two-factor model^d^	2507.33	229	0.69	0.67	0.69	0.14	0.17
One-factor model^e^	3656.67	230	0.54	0.52	0.54	0.16	0.21

*n = 336. ^*a*^OBSE and PIS were combined. ^*b*^OBSE and PIS were combined, IRB and OCB were combined. ^*c*^Hostile attribution bias, OBSE and PIS were combined, IRB and OCB were combined. ^*d*^Perceived negative workplace gossip, hostile attribution bias, OBSE and PIS were combined, and IRB and OCB were combined. ^*e*^All variables were combined into a single factor.*

### Descriptive Statistics

Means, standard deviations, and correlations of variables in the study are presented in **Table 2**. Perceived negative workplace gossip is significantly correlated with employees’ IRB (*r* = -0.28, *p*<0.01), OCB (*r* = -0.26, *p*<0.01), OBSE (*r* = -0.29, *p*<0.01), and PIS (*r* = -0.23, *p*<0.01) as well. Moreover, OBSE is significantly correlated with employees’ IRB (*r* = 0.34, *p*<0.01) and OCB (*r* = 0.29, *p*<0.01), and PIS is significantly correlated with employees’ IRB (*r* = 0.22, *p*<0.01) and OCB (*r* = 0.21, *p*<0.01).

**Table 2:** Means, standard deviations, and correlations.

	*M*	*SD*	1	2	3	4	5	6	7	8	9	10
1. Gender^a^	0.57	0.51										
2. Age	28.94	2.94	0.00									
3. Education	1.28	0.45	0.12*	–0.07								
4. Organizational tenure	6.08	2.75	–0.01	0.68**	–0.10							
5. Years with the leader	2.23	1.69	–0.16**	0.38**	0.04	0.35**						
6. Perceived negative workplace gossip	1.71	0.58	0.09	0.09	–0.04	0.11	–0.08					
7. Hostile attribution bias	2.66	0.52	0.06	0.13*	–0.05	0.19**	0.07	**0.18^∗∗^**				
8. OBSE	3.62	0.60	0.08	–0.08	0.27**	–0.20**	0.05	–**0.29^∗∗^**	–**0.14^∗^**			
9. PIS^b^	22.28	4.77	–0.05	–0.05	0.12*	–0.16**	–0.01	–**0.23^∗∗^**	–**0.22^∗∗^**	**0.39^∗∗^**		
10. IRB	3.86	0.56	0.03	–0.18**	0.07	–0.20**	–0.129*	–**0.28^∗∗^**	–**0.21^∗∗^**	**0.34^∗∗^**	**0.22^∗∗^**	
11. OCB	3.89	0.40	0.01	–0.19**	0.12*	–0.15**	–0.05	–**0.26^∗∗^**	–**0.20^∗∗^**	**0.29^∗∗^**	**0.21^∗∗^**	**0.34^∗∗^**

*n = 336; ^∗∗^*p* < 0.01, ^∗^*p* < 0.05. ^*a*^Gender: 0(female), 1(male). ^*b*^The scale score was calculated by summing the individual item scores.*

### Hypothesis Testing

Hypotheses 1 predicts that perceived negative workplace gossip negatively influences employees’ IRB and OCB. The analysis result shown in **Table 3** indicates that perceived negative workplace gossip produces a significant negative impact on employees’ IRB (M2, β = -0.28, *p* < 0.01) and OCB (M8, β = -0.24, *p* < 0.01). Therefore, Hypotheses 1a and 1b were supported.

**Table 3:** Results of mediating effect analysis.

	In-role behavior						Organizational citizenship behavior					
	M1	M2	M3	M4	M5	M6	M7	M8	M9	M10	M11	M12
Gender	0.01	0.03	–0.01	0.01	0.02	0.04	0.00	0.02	–0.02	0.00	0.01	0.02
Age	–0.07	–0.05	–0.10	–0.08	–0.09	–0.07	–0.16	–0.14*	–0.18*	–0.17*	–0.18*	–0.16
Education	0.06	0.05	–0.02	–0.02	0.03	0.03	0.11	0.10	0.04	0.05	0.09	0.08
Organizational tenure	–0.12	–0.09	–0.03	–0.02	–0.08	–0.06	–0.03	–0.01	0.04	0.04	0.01	0.02
Years with the leader	–0.06	–0.10	–0.10	–0.12	–0.06	–0.09	0.02	–0.01	–0.02	–0.03	0.02	–0.01
PNWG		–**0.28^∗∗^**		–**0.20^∗∗^**		–**0.25^∗∗^**		–**0.24^∗∗^**		–**0.18^∗∗^**		–**0.21^∗∗^**
OBSE			**0.34^∗∗^**	**0.28^∗∗^**					**0.28^∗∗^**	**0.23^∗∗^**		
PIS					**0.20^∗∗^**	**0.14^∗∗^**					**0.19^∗∗^**	**0.14^∗^**

*R*^2^	0.03	0.11	0.14	0.17	0.07	0.09	0.03	0.09	0.10	0.13	0.06	0.10
*F*	3.39**	7.68**	9.68**	10.71**	5.19**	6.41**	3.26**	6.35**	7.15**	7.93**	4.80**	6.49**

*n = 336; ^∗∗^*p* < 0.01, ^∗^*p* < 0.05. PNWG, perceived negative workplace gossip; OBSE, organizational-based self-esteem; PIS, perceived insider status.*

Hypothesis 2 proposes that the relationship between perceived negative workplace gossip and employees’ behaviors is mediated by OBSE and PIS. **Tables 3, 4** show that: first, with demographic variables controlled, perceived negative workplace gossip has a significant negative correlation with OBSE (M14, β = -0.27, *p* < 0.01) and PIS (M17, β = -0.22, *p* < 0.01); next, after the entry of OBSE and PIS into regression, OBSE (M4, β = 0.28, *p* < 0.01) and PIS (M6, β = 0.14, *p* < 0.01) are significantly related to employees’ IRB while the effect of perceived negative workplace gossip on employees’ IRB (M4, β = -0.20, M6, β = -0.25, *p* < 0.01) is all reduced. By combining these results, we have found the initial support for the mediating role of OBSE and PIS (Baron and Kenny, 1986; Mathieu and Taylor, 2007). In order to further examine the mediating effect, we also adopted Sobel (1982) test. The results of Sobel test demonstrate significant mediating effect (*Z* = -4.12 for OBSE and *Z* = -2.78 for PIS, *p* < 0.01). Therefore, Hypotheses 2a is supported.

**Table 4:** Results of moderating effect analysis.

	OBSE			PIS		
	M13	M14	M15	M16	M17	M18
Gender	0.06	0.08	0.09	–0.07	–0.05	–0.04
Age	0.08	0.10	0.08	0.10	0.12	0.10
Education	0.24**	0.23**	0.24**	0.12*	0.11*	0.11*
Organizational tenure	–0.27**	–0.24**	–0.20**	–0.22**	–0.20**	–0.15*
Years with the leader	0.12	0.08	0.09	0.01	–0.02	–0.01
Perceived negative workplace gossip		–**0.27^∗∗^**	–**0.29^∗∗^**		–**0.22^∗∗^**	–**0.21^∗∗^**
Hostile attribution bias			–**0.07**			–**0.16^∗∗^**
Negative workplace gossip ^∗^ hostile attribution bias			**0.24^∗∗^**			**0.14^∗∗^**
*R*^2^	0.11	0.18	0.23	0.03	0.08	0.11
*F*	9.09**	12.97**	13.69**	3.20**	5.60**	6.29**

*n = 336; ^∗∗^*p* < 0.01, ^∗^*p* < 0.05. OBSE, organizational-based self-esteem; PIS, perceived insider status.*

What is more, after the entry of OBSE and PIS into regression, OBSE (M10, β = 0.23, *p* < 0.01) and PIS (M12, β = 0.14, *p* < 0.01) are significantly related to employees’ OCB while the effect of perceived negative workplace gossip on employees’ OCB (M10, β = -0.18, M12, β = -0.21, *p* < 0.01) is all reduced. By combining these results, we have found the initial support for the mediating role of OBSE and PIS (Baron and Kenny, 1986; Mathieu and Taylor, 2007). The results of Sobel test demonstrate significant mediating effect (*Z* = -4.28 and -2.48, *p* < 0.05). Therefore, Hypotheses 2b is supported.

**FIGURE 2:**
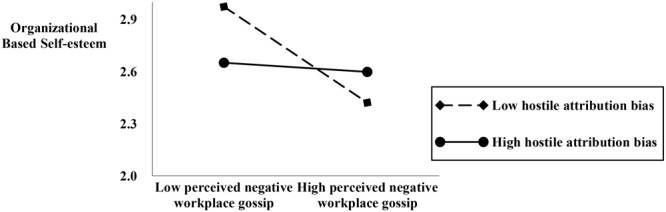
The moderating effect of hostile attribution bias.

**FIGURE 3:**
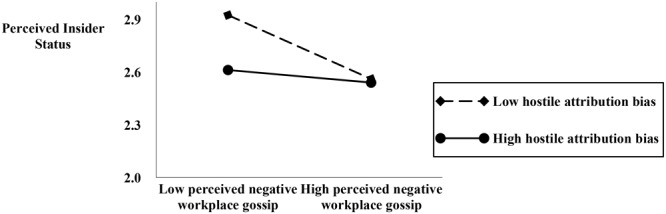
The moderating effect of hostile attribution bias.

Hypothesis 3 proposes that hostile attribution bias moderates the relationship between perceived negative workplace gossip and self-concept (OBSE and PIS). As indicated by M15 and M18 in **Table 4**, the interactive effect is 0.24 (*p* < 0.01) and 0.14 (*p* < 0.01). Thus, Hypothesis 3 is supported. Following the procedures recommended by Aiken and West (1991), we charted and conducted the simple slope test. As shown in **Figures 2, 3**, for employees with high level of hostile attribution bias, perceived negative workplace gossip establishes a stronger negative relationship with OBSE and PIS. Thus, Hypothesis 3 is supported.

Hypothesis 4 proposes two moderated-mediation models. Moderated path analysis approach (bootstrapping method, Edwards and Lambert, 2007) was applied to estimate two set of effects at high and low levels of moderators.

As predicted by H4a, hostile attribution bias moderates the mediating effect of OBSE and PIS in the relationship between perceived negative workplace gossip and employees’ IRB. As indicated in **Tables 5, 6**, hostile attribution bias has more significant moderating effect in the first stage (β = 0.34 for OBSE and β = 1.64 for PIS, *p* < 0.05) than the second stage (β = -0.16 for OBSE and β = -0.02 for PIS, *ns*). However, the indirect effect of perceived negative workplace gossip on employees’ IRB via OBSE (β = 0.13, *p* < 0.05) and PIS (β = 0.07, *p* < 0.05) is significantly moderated. What is more, CI excluded the zero value (95% CI = 0.011–0.276 for OBSE and 95% CI = 0.016–0.164 for PIS), indicating the significance of the indirect relationships. Thus, H4a is supported.

**Table 5:** Results of the moderated path analysis.

	Perceived negative workplace gossip → OBSE → IRB				
Hostile attribution bias	Stage		Effect		
	Stage 1	Stage 2	Direct	Indirect	Total
Low hostile attribution bias (-1 s.d.)	–0.49**	0.32*	–0.27**	–0.16**	–0.42**
High hostile attribution bias (+1 s.d.)	–0.15*	0.16*	–0.10	–0.03	–0.12
Difference between high and low	0.34*	–0.16	0.17	0.13*	0.30**

*n = 336; ^∗∗^*p* < 0.01, ^∗^*p* < 0.05. OBSE, organizational-based self-esteem; IRB, in-role behavior.*

**Table 6:** Results of the moderated path analysis.

	Perceived negative workplace gossip → PIS → IRB				
Hostile attribution bias	Stage		Effect		
	Stage 1	Stage 2	Direct	Indirect	Total
Low hostile attribution bias (-1 s.d.)	–2.63**	0.03*	–0.32**	–0.08*	–0.40**
High hostile attribution bias (+1 s.d.)	–0.99*	0.01	–0.12	–0.01	–0.13
Difference between high and low	1.64*	–0.02	0.20	0.07*	0.27*

*n = 336; ^∗∗^*p* < 0.01, ^∗^*p* < 0.05. PIS, perceived insider status; IRB, in-role behavior.*

As predicted by H4b, hostile attribution bias moderates the mediating effect of OBSE and PIS in the relationship between perceived negative workplace gossip and employees’ OCB. As indicated in **Tables 7, 8**, hostile attribution bias has more significant moderating effect in the first stage (β = 0.34 for OBSE and β = 1.64 for PIS, *p* < 0.05) than the second stage (β = -0.09 for OBSE and β = -0.02 for PIS, *ns*). However, the indirect effect of perceived negative workplace gossip on employees’ OCB via OBSE (β = 0.09, *p* < 0.05) and PIS (β = 0.05, *p* < 0.05) is significantly moderated. What is more, CI excluded the zero value (95% CI = 0.004–0.157 for OBSE and 95% CI = 0.005–0.126 for PIS). Thus, H4b is supported.

**Table 7:** Results of the moderated path analysis.

	Perceived negative workplace gossip → OBSE → OCB				
Hostile attribution bias	Stage		Effect		
	Stage 1	Stage 2	Direct	Indirect	Total
Low hostile attribution bias (-1 s.d.)	–0.49**	0.19*	–0.19**	0.10**	–0.28**
High hostile attribution bias (+1 s.d.)	–0.15*	0.10	–0.05	–0.01	–0.07
Difference between high and low	0.34*	–0.09	0.14	0.09*	0.21**

*n = 336; ^∗∗^*p* < 0.01, ^∗^*p* < 0.05. OBSE, organizational-based self-esteem; OCB, organizational citizenship behavior.*

**Table 8:** Results of the moderated path analysis.

	Perceived negative workplace gossip → PIS → OCB				
Hostile attribution bias	Stage		Effect		
	Stage 1	Stage 2	Direct	Indirect	Total
Low hostile attribution bias (-1 s.d.)	–2.63**	0.02*	–0.21**	–0.05*	–0.26**
High hostile attribution bias (+1 s.d.)	–0.99*	0.00	–0.07	0.00	–0.07
Difference between high and low	1.64*	–0.02	0.14	0.05*	0.19**

*n = 336; ^∗∗^*p* < 0.01, ^∗^*p* < 0.05. PIS, perceived insider status; OCB, organizational citizenship behavior.*

## Discussion

In organizations, gossips serve as a major tool to strengthen informal employee relationship (Noon and Delbridge, 1993; Dunbar, 2004; Kniffin and Wilson, 2005). Although negative workplace gossip means social undermining to employees (Duffy et al., 2002), almost everyone in the workplace is creating, listening to, and discussing it (Foster, 2004). The study mainly explores the impact of perceived negative workplace gossip on employees’ behaviors and its process from the self-evaluation perspective. 336 employees from nine Chinese companies in diverse industries and with various job types were investigated for empirical analysis on proposed hypotheses, and results show that: (1) Perceived negative workplace gossip adversely influences employees’ IRB and OCB. (2) Self-concept (OBSE and PIS) plays a mediating role in the relationship between perceived negative workplace gossip and employees’ behaviors (IRB and OCB). (3) Employees’ hostile attribution bias moderates the relationship between perceived negative workplace gossip and self-concept (OBSE and PIS). (4) Employees’ hostile attribution bias moderates the mediating effect of self-concept (OBSE and PIS) on the relationship between perceived negative workplace gossip and employees’ behaviors (IRB and OCB).

### Theoretical Significance

Firstly, the study represents pioneering efforts to empirically investigate the impact of consequences of negative workplace gossip in Chinese cultural background, which enriches and expands researches on negative workplace gossip. Specifically, negative workplace gossip has gradually become a hotspot in the research field of organizational behavior (Foster, 2004; Waddington and Michelson, 2007; Feinberg et al., 2014), but little study or exploration can be found in Chinese academic society. Based on the self-evaluation perspective, the paper conducts research on the relationship between negative workplace gossip and employees’ behaviors, and gives explanations based on the contingency perspective, benefiting domestic scholars’ deep understanding of negative workplace gossip. Besides, the paper starts from the negative factor of negative workplace gossip, and makes discussion on the influence factor of employees’ behaviors, significantly supplementing researches on employees’ behaviors.

Secondly, our study also helps advance the self-verification theory. Scholars propose that self-verification, as one of the most important human motivations (Kwang and Swann, 2010; Swann, 2011), can bring individuals such benefits as enhancement of control sense and predictive power of the outside (Swann, 1987). Such theory helps improve our understanding of the process (OBSE and PIS) through which perceived negative workplace gossip influences target behavior (IRB and OCB), and the boundary condition of the hostile attribution bias. One possible reason that the literature on workplace gossip is being held back is that it lacks a theoretical framework (Wu et al., 2016). Therefore, findings of this research contribute to knowledge on the interpersonal effects of information spread based on self-verification theory, especially on the relationship between negative gossip and positive behaviors.

Third, the study also reveals the importance of OBSE and PIS. The paper starts from self-verification theory to explore the mediating mechanism of negative workplace gossip. The results show that negative workplace gossip exerts negative influence on employees’ behaviors through the mediating effect of OBSE and PIS. Meanwhile, withholding OBSE and PIS of employees by negative workplace gossip implies that employees’ OBSE and PIS is affected by not only structural factors (such as organization structure and work design) and personal work experience or achievements but also employees’ interpersonal interaction with colleagues. The conclusion agrees with viewpoints of interpersonal interactionism in self-verification theory: employees negatively evaluated by others in an organization will integrate negative evaluation into his/her self-evaluation, which will affect employees’ OBSE and PIS, and then self-verify his/her OBSE and PIS (Korman, 1970; Swann, 1987).

Last but not least, the study extends researches on negative organizational behavior through self-verification theory (Korman, 1970; Swann, 1987, 2011). Self-verification theory argues that an individual tends to perform behaviors or attitudes consistent with his/her self-concept or self-evaluation so as to verify his/her self-concept or self-evaluation (such as esteem). Scholars propose that self-verification, as one of the most important human motivations (Kwang and Swann, 2010; Swann, 2011), can bring individuals such benefits as enhancement of control sense and predictive power of the outside (Swann, 1987). In front of negative organizational behaviors (such as negative workplace gossip), individuals with negative self-evaluation are inclined to self-verify negative self-evaluation, and perform a series of negative attitudes and behaviors, etc. Achievements of this study have manifested the theory content, and enriched theory research in the field of negative organizational behaviors.

### Practical Implications

People personally process and modify disseminating gossips based on their own wish, habit, concern, prejudice, and expectation, and not a few people will randomly change gossip contents. Once disseminated, gossips will become more and more weird and twisted – “many times of dissemination will make black and white reversed.” Hence, the study on negative workplace gossip is of great significance for practice.

First, the study demonstrates that negative workplace gossip can significantly withhold OBSE and PIS, and then reduce employees’ workplace behaviors. Therefore, organizations should take measures to handle negative workplace gossip: (1) Advocate interaction equality and emphasize employees’ cognition of dignity and respect. Organizations should make efforts to establish and maintain fair interaction so as to ensure employees’ dignity and respect. (2) Establish effective communication channels. As mentioned above, reasons for the existence of “little broadcast” and “hearsay” are that human beings make social contact, and like small talks with some curious or privately informative features. In particular within an organization with unsmooth formal channels, each member masters some inconsistent information, which makes negative gossip spread more quickly (Dunbar et al., 1997; Foster, 2004). Therefore, effective methods for eliminating negative workplace gossip are to perfect formal and informal communication channels and guarantee channel smoothness.

Second, the study finds that OBSE and PIS can positively drive employees’ behaviors. That is to say, one of effective methods to improve employees’ behaviors and attitudes is to enhance employees’ OBSE and PIS. OBSE reflects employees’ self-sensed value in organizations, PIS reflects an employees’ perception of membership in a certain organization. Studies have proven that organizational identification, sense of trust, perception of organizational fairness, well-defined role, task complexity, job security, etc. can all exert significant influence on OBSE and PIS, so employees’ OBSE and PIS can be enhanced by improving their organizational identification, strengthening leader–employee trust, increasing perception of organizational fairness, etc., so as to reduce negative influence of negative workplace gossip on employees and adding positive outputs.

Last, hostile attribution bias plays a major role of catalyst for the destructive function of negative workplace gossip. Hostile attribution bias is one of important individual traits. Individuals with high hostile attribution bias are quite sensitive to organizational context, no matter for good or bad signals. The study indicates that hostile attribution bias strengthens the effect of negative workplace gossip on IRB and OCB via OBSE and PIS, and its enlightenment on practice is that organizations should try to release clear explanation on ambiguous context information from system and operation levels, so as to resist employees from negatively evaluating ambiguous information. What is more, managers, in particular direct leaders of employees with high hostile attribution bias, should advocate a positive organizational atmosphere, and as long as negative organizational behaviors occur, pay special attention to these employees and offer positive guidance.

### Strengths and Limitations

One important advantage of the study is its preciseness of design: It adopted longitudinal study design with multiple points in time to avoid common method variance and make research conclusions more realistic and reliable. However, certain limitations also exist: (1) Most scales in the study were developed in the western organizational background, and although the measurement stability of some scales has been proven among Chinese and western samples, local or localized scales will make research conclusions closer to local reality; (2) Enterprises involved in the study were selected by the researchers on their own, restricting the study’s external validity to a certain degree, so follow-up studies should make investigation in a wider range. (3) This study discussed the independent variable of employees’ behavioral only from negative workplace gossip. The future research can also be studied from the perspective of the economic crisis, because this is a new trend in the current period and need to be addressed. (4) Besides, the paper discusses the mediating effect mechanism of OBSE and PIS between negative workplace gossip and employees’ behavior, but there may be different paths for negative workplace gossip to affect employees’ work behavior and attitude, so future studies can apply different theoretical perspectives to explore the mediating effect mechanism of negative workplace gossip. For example, social exchange theory is drawing more and more attention from academic world to explain employees’ work behavior and attitude, and leader’s style can also exert important influence on employees’ attitude and behavior. (5) As mentioned above, negative workplace gossip is complicated, and not all negative gossips lead to bad results, so exploration on distinguishing the functions of negative workplace gossip can be considered from organizational perspective and employees’ perspective (Grosser et al., 2012). The paper only makes analysis from employees’ perspective, and follow-up studies can explore specific functional mechanism from organizational perspective to find whether negative workplace gossip beneficial to organization exists and how such gossips play a role and can be effectively controlled. These questions can all be research questions with both theoretical and practical significance.

## Conclusion

The study mainly explores the impact of perceived negative workplace gossip on employees’ behaviors and its process from the self-evaluation perspective. 336 employees from nine Chinese companies in diverse industries and with various job types were investigated for empirical analysis on proposed hypotheses, and results show that: Perceived negative workplace gossip adversely influences employees’ IRB and OCB differentially by changing employees’ self-concept (OBSE and PIS). Further, the relative impact of either type behaviors depends on employees’ hostile attribution bias. Thus, the findings of this research offer deeper insights into the potential harms of gossips and contribute to outlining the underlying mechanism and boundary condition of “perceived negative workplace gossip-employees’ behaviors” linkage.

## Author Contributions

The author confirms being the sole contributor of this work and approved it for publication.

## Conflict of Interest Statement

The authors declare that the research was conducted in the absence of any commercial or financial relationships that could be construed as a potential conflict of interest.
