# Corrigendum: Effect of Perceived Negative Workplace Gossip on Employees' Behaviors

**DOI:** 10.3389/fpsyg.2018.02728

**Published:** 2019-01-11

**Authors:** Ming Kong

**Affiliations:** School of Management, Shandong University, Jinan, China

**Keywords:** perceived negative workplace gossip, in-role behavior, organizational citizenship behavior, organization-based self-esteem, perceived insider status, hostile attribution bias

In the original article, we neglected to include the funder “National Natural Science Foundation of China, 71872102.” The updated funding statement is mentioned below:

This research was supported by National Natural Science Foundation of China (No. 71872102).

The authors apologize for this error and state that this does not change the scientific conclusions of the article in any way. The original article has been updated.

